# Activated Ras as a Therapeutic Target: Constraints on Directly Targeting Ras Isoforms and Wild-Type versus Mutated Proteins

**DOI:** 10.1155/2013/536529

**Published:** 2013-10-31

**Authors:** Raymond R. Mattingly

**Affiliations:** Department of Pharmacology, Wayne State University, 540 East Canfield Avenue, Detroit, MI 48201, USA

## Abstract

The ability to selectively and directly target activated Ras would provide immense utility for treatment of the numerous cancers that are driven by oncogenic Ras mutations. Patients with disorders driven by overactivated wild-type Ras proteins, such as type 1 neurofibromatosis, might also benefit from progress made in that context. Activated Ras is an extremely challenging direct drug target due to the inherent difficulties in disrupting the protein:protein interactions that underlie its activation and function. Major investments have been made to target Ras through indirect routes. Inhibition of farnesyl transferase to block Ras maturation has failed in large clinical trials. Likely reasons for this disappointing outcome include the significant and underappreciated differences in the isoforms of Ras. It is still plausible that inhibition of farnesyl transferase will prove effective for disease that is driven by activated H-Ras. The principal current focus of drugs entering clinic trial is inhibition of pathways downstream of activated Ras, for example, trametinib, a first-in-class MEK inhibitor. The complexity of signaling that is driven by activated Ras indicates that effective inhibition of oncogenic transduction through this approach will be difficult, with resistance being likely to emerge through switch to parallel pathways. Durable disease responses will probably require combinatorial block of several downstream targets.

## 1. Introduction: Ras Activation and Cancer

Ras proteins are key controllers of cellular growth and differentiation [1], with critical roles in the development [2, 3] and maintenance [4] of human tumors. As the prototypical small GTPase, Ras is regulated through an activation/deactivation cycle of exchange of GTP for GDP and subsequent GTP hydrolysis [5]. The GTP-bound state is the active conformation that can couple to downstream effectors [6]. The slow intrinsic rates of activation and deactivation of wild-type Ras allow catalytic control through exchange factors (GEFs) and GTPase-activating proteins (GAPs) [7]. Although acute decrease in GAP activity was the first mechanism described for agonist-induced Ras activation [8, 9], most instances of acute Ras activation are probably due to regulation of GEF activity [10, 11]. 

About 30% of human cancers have a mutated Ras protein [12] that is constitutively bound to GTP [13] due to decreased GTPase activity and insensitivity to GAP action [14–16]. Ras is also an important factor in many cancers where it is not mutated but rather functionally activated through inappropriate activity of other signal transduction elements, for example, by overexpressed growth factor receptors in breast cancer [17, 18] or by loss of a GAP, such as in type 1 neurofibromatosis (NF1) [19]. Thus there are at least three distinct routes to inappropriate Ras activation in cancer: (1) mutational activation of Ras itself, (2) excessive activation of the wild-type protein through upstream signaling leading to increased GEF activity, and (3) loss of a GAP function that is required to terminate activity of the wild-type protein. There are continuing efforts to understand whether there are significant differences in the activation state of Ras that is the result of these distinct mechanisms [20, 21].

There are four human Ras proteins (H-Ras, 2 splice variants of K-Ras, and N-Ras). Although their effector domains are identical, the isoforms clearly have some distinct and nonoverlapping functions [22, 23]. For example, only K-Ras is essential during mouse development [24]. From the perspective of human disease it is most notable that K-Ras is mutated at high prevalence in particular forms of cancer, notably pancreatic, lung, and colon carcinomas, while mutated N-Ras is found at high rates in melanomas and some leukemias, and mutated H-Ras is a rarer finding that is present in some bladder, breast, and thyroid carcinomas [25]. The main structural differences between the isoforms occur in the short hypervariable region just prior to their C-termini [26], and many groups have localized isoform-specific functions to this region [27–35]. The fact that oncogenic K-Ras function can be shown to depend on the endogenous activity of wild-type H- or N-Ras in at least some systems [27, 36] emphasizes the importance of understanding routes to inhibition of all the isoforms and both the mutated and intact states of Ras.

The Ras isoforms exhibit particular subcellular localizations [37, 38] that contribute both to the *in situ *selectivity of their activation and deactivation [39] and to isoform-specific downstream signaling [40]. It is not entirely clear whether the limited biochemical and cell biological distinctions that are known to exist between these extremely similar proteins provide a full explanation for the striking functional differences found in the involvement of the isoforms in human disease or if there may be additional factors that are still to be elucidated. For example, in addition to differences at the protein level it is possible that there could be significant isoform selectivity exerted through processes such as microRNA regulation [41–43].

## 2. Approaches to Directly Inhibit Activated Ras

The identification of oncogenically mutated Ras in many human cancers led to major efforts in many academic laboratories and large and small drug companies to target this constitutively activated protein as a rational and selective treatment. There are several very significant challenges that have confronted these investigators and there has yet to be an effective drug with a mechanism of inhibition of activated Ras that has shown sufficient success in clinical trials to achieve approval for use in humans. It is notable that many of the effective drugs in current use are either natural products [44], most often botanicals, or semisynthetic/synthetic analogs thereof, and that about half of all drugs target G protein-coupled receptors by competing at the endogenous ligand binding site that is exposed at the cell surface [45]. Ras does not have the characteristics that define these classes of premier drug targets. The only natural products that are known to target it are bacterial toxins, such as large clostridial cytotoxins, and these act enzymatically to produce a covalently modified Ras that is resistant to GAP [46]. They thus do not provide a precedent that would help to guide the development of potentially useful small molecules that would restore GTPase activity. Further, Ras proteins are intracellular enzymes. Although small molecules can be designed to block many active sites, the problem presented by mutated Ras is due to loss of enzymatic activity (decreased GTPase function) that leads to a phenotypic gain of function (increased signaling activity). Thus, a small molecule that functioned to block the active site of Ras would be the opposite of what was desired and might presumably be oncogenic. The challenge of restoring enzymatic function to an impaired active site by use of a small molecule is very difficult. In the case of mutationally activated Ras proteins, landmark structural studies indicated that it may not be feasible to produce a small molecule that could replace the loss of GAP or GTPase activity that is the molecular mechanism driving the dysfunction [47]. 

The result of the loss of GAP or GTPase activity is that the Ras protein is inappropriately bound to GTP at too high a proportion and for too long a time. It was reasonable to speculate, therefore, that perhaps a small molecule could be designed to displace the GTP from the Ras protein, with encouragement provided by the ability of drugs to work competitively against the ATP binding site of protein kinases. The explosion of work in that area moved protein kinases into position as the second largest group of potential drug targets after the G protein-coupled receptors [48]. Several druggable routes to kinase inhibition have been identified, with competition for ATP binding at the active site being the predominant molecular mechanism [49]. Unfortunately, however, competition for the nucleotide binding site is not a promising approach for targeting activated Ras due to the kinetic parameters of Ras:GTP binding, which is characterized by a very high affinity (picomolar) in the context of millimolar cytosolic GTP levels [50]. The situation of protein kinase inhibition is distinct because the affinity for ATP is typically micromolar in the context of millimolar cytosolic nucleotide pools and thus the nanomolar affinity that can be achieved with a good small molecule inhibitor is sufficient to be efficacious [51].

The result of Ras being bound to GTP is that it forms complexes with its downstream targets to drive oncogenic signaling. Disruption of intracellular protein:protein interactions is not an established approach for therapeutic targeting, but *in vitro *experiments using peptides and peptidomimetic agents have produced interesting results [52, 53]. The outstanding possibilities for new targets that would be revealed by such targeting, which could include transcription factors, were recognized by the National Cancer Institute in the form of a request for applications on this topic in their “provocative questions” series (http://provocativequestions.nci.nih.gov/archived-rfas-and-pqs/rfa-archive-2011/mainquestions_listview?mqCategory=Treatment). Despite some early progress [54], the Ras:effector interaction was characterized as “undruggable” as recently as 2010 in a study that demonstrated the potential value of this intervention by expression of a blocking antibody fragment [55]. There has been continuing interest in whether Ras:effector binding could be susceptible to drug inhibition, culminating in a new report that provides significant and long awaited progress in this area [56]. The authors demonstrate small molecules that can block the binding of H-Ras.GTP to c-Raf1 both *in vitro* and *in vivo*, that inhibit multiple Ras-driven pathways in cells, and that are orally active against a K-Ras mutation positive colon carcinoma xenograft. 

There has also been recent progress in disruption of Ras protein:protein interactions at the level of its interaction with one of its upstream activator GEF proteins, Sos [57], including development of small molecule agents [58, 59]. Both of these latter studies began with an activated K-RasG12D construct, whereas the former investigated a system of wild-type Ras activation by upstream receptor tyrosine kinases. In this context it is worthwhile to consider the relative degrees to which mutated versus wild-type Ras proteins are reliant on interaction with GEFs in order to achieve the GTP-bound state [36]. For example, it is likely that wild-type Ras proteins would be significantly more dependent on sustained GEF activity to maintain their activation state and so could be more vulnerable to targeting at this level [56]. Another aspect to consider is the degree to which inhibitors of interactions between small GTPases and their GEFs will only block the stimulated rate of nucleotide exchange [58] or also affect the intrinsic rate of exchange [60]. Overall, we do not yet know whether the same constraints and opportunities will apply to targeting wild-type Ras proteins that are being driven by upstream oncogenic signaling and to targeting mutationally activated Ras proteins. 

## 3. Selection of Farnesyl Transferase as the Primary Target for Indirect Inhibition of Activated Ras

The absence of an obvious direct druggable target at the level of activated Ras itself led to a broader review of Ras biology to find an appropriate druggable enzyme. Ras isoforms are translated as soluble proteins on free ribosomes and then undergo a series of posttranslational modifications to achieve membrane attachment and the localization that is required for biological function. The first reaction is prenylation and is catalyzed by protein farnesyl transferase (FTase), with subsequent proteolysis and methylation modifications. FTase binds to proteins bearing a carboxyl-terminal motif referred to as a “CaaX box,” where C means cysteine, a means any residue (typically aliphatic), and X means serine, methionine, glutamine, or alanine [61] and transfers a farnesyl group from farnesyl pyrophosphate (FPP) to the sulfur on the cysteine side chain. A closely related enzyme catalyzes an alternative prenylation reaction. Protein geranylgeranyl transferase I (GGTase I) attaches a geranylgeranyl moiety from geranylgeranyl diphosphate (GGPP) to the cysteine in a similar CaaX box in different proteins, primarily those where leucine is the carboxyl-terminal residue. The prenylated protein (whether farnesylated or geranylgeranylated) then undergoes endoproteolytic cleavage of the aaX residues by the enzyme Rce1 and methylation of the resulting free carboxyl group by the enzyme isoprenylcysteine carboxyl methyltransferase (Icmt). The overall effect of these modifications is to convert a soluble peptide or protein into a hydrophobic, membrane-bound species. These posttranslational changes facilitate association with lipid membranes but also govern subcellular localization and regulate protein:protein interactions [62–64]. In addition to these steps, N-Ras, H-Ras, and K-Ras4A isoforms are further modified with the adjacent attachment of palmitate [65]. K-Ras4B is not palmitoylated. A polylysine stretch of amino acids in the hypervariable region of this isoform confers additional targeting and association for its membrane localization [65]. 

The demonstration that posttranslational covalent modification of the CaaX box is essential for the transforming activity of Ras [66] provided the rationale that inhibiting the various enzymes responsible for this processing would suppress the transforming activity of mutant Ras. Presumably, improper posttranslational modification would lead to mislocalized and thus nonfunctional protein [67]. Further, it was conjectured that mislocalized, constitutively-active Ras might recruit and sequester signaling molecules to inappropriate subcellular locations and so act as an inhibitor of Ras-driven signaling in tumor cells [68]. If this scenario were realized then there could be some selective toxicity for cancer cells over other cells that only express wild-type Ras proteins. 

The major focus of most therapeutic development for inhibition of Ras so far has thus been to block FTase, using farnesyl transferase inhibitors (FTIs) [69]. The intense activity in this area was spurred by the dramatic tumor regressions seen in experimental animals with FTIs [70] and many potent FTIs of divergent structures were developed [71–73]. Two FTIs made it all the way to phase III clinical trials—R115777/Zarnestra/tipifarnib [74] and SCH66336/Sarasar/lonafarnib [75]—before it was confirmed that they lack the anticipated activity against lung, colorectal, and pancreatic cancer [76]. 

## 4. Disappointment with FTIs in the Clinic: The Problem of Alternative Prenylation of K- and N-Ras

In retrospect, even before the large-scale clinical trials, many tumor cells that were positive for K-Ras mutations had been shown to be resistant to FTIs [77]. Several factors may explain why FTIs are not as useful in the clinic as had been hoped. A major reason is probably that they were developed primarily using mutated H-Ras as an experimental target. Despite early assumptions that all the Ras isoforms would be similarly affected, only H-Ras is efficiently perturbed in FTI-treated tumors [78, 79]. N- and K-Ras are also *in vitro* substrates for GGTase I [80] and become geranylgeranylated in cells in the presence of FTIs [81, 82]. This alternative prenylation presumably allows N- and K-Ras to continue their maturation steps and become functional and thus circumvent the action of FTIs [61]. 

There has been some consideration of whether it could be possible to render N- and K-Ras sensitive to inhibition of prenylation, with demonstration of some encouraging results *in vitro*. Limitation of cellular prenyl substrate pools by treatment with statins (inhibitors of HMG CoA reductase) can potentiate the action of FTIs [83, 84]. This potentiation can be synergistic for combinations of statins with FTIs that are competitive with the FPP substrate of the enzyme [85–87]. A rationale for this combination approach of prenylation inhibitors [statin plus an FPP-competitive FTI] is to produce a block of FTase plus limitation of cellular pools of prenyl precursors. Decreased levels of FPP will intensify the FTase inhibition and decreased levels of GGPP will blunt the ability of N-Ras or K-Ras to become alternatively geranylgeranylated [86, 88]. Combinations of statin plus GGTase inhibitors also act synergistically to block prenylation [89]. It is possible that the benefit of the combination approach with statins may not be restricted to FTIs that are competitive with FPP. For example, a combination of tipifarnib (a CaaX-competitive inhibitor) plus lovastatin can effectively block N-Ras and K-Ras processing in multiple myeloma cells [90]. Whether this combination approach of statin + FTI would achieve a clinical benefit has not yet been tested. 

## 5. Failure of FTase as a Therapeutic Target: Is There Still Promise for Treatment of H-Ras-Driven Disease?

The initial hope had been that FTIs would be effective drugs to treat cancers such as lung, colon, and pancreatic carcinomas that are often driven by mutated K-Ras and afflict huge numbers of patients. Their lack of efficacy in these diseases has focused attention on our current lack of ability to selectively inhibit oncogenic K-Ras [12, 91]. Similarly, mutant N-Ras that drives a significant fraction of malignant melanomas and some leukemias remains problematic despite the recent encouraging results with MEK inhibition as a downstream intervention [92], which is discussed further below. 

On the other hand, and in view of the immense investment that has been put into the development and testing of FTIs, it is perhaps surprising that they do not seem to have been systematically tested in cancers that are driven by mutated H-Ras. Although much smaller in number, patients do present with cancers that have oncogenic H-Ras mutations and the available evidence would strongly suggest that FTIs could be a rational approach to their disease. With the increasing use of personalized medicine that will include profiling of the mutation status of tumors that will be coupled to pathway profiling to identify driving oncogenic mechanisms [93], it is possible that FTIs will eventually produce benefits in patients with disease that is driven through oncogenic H-Ras function. A recent analysis of the COSMIC database of human cancers indicates that about 3% of cases may harbor mutated H-Ras [12].

## 6. Role of H-Ras Activation in Learning and Memory

One complication with targeting Ras function for treatment of cancer will be if there are essential normal functions of the protein that would also be disrupted. In this regard it is important to note an emerging model, which is supported by human genetic disorders that result in learning deficits, that has Ras activation as a critical part of the synaptic remodeling process that underlies memory [94]. For example, truncation mutations of SYNGAP1, a regulator of Ras in neurons, are found in patients with nonsyndromic mental retardation [95]. At the mechanistic level, calcium-dependent Ras activation in neurons [96] is at least part of the calmodulin-dependent protein kinase II (CaMKII) mediated mechanism [97] through which excitatory, NMDA-type glutamate receptors lead to synaptic plasticity [98]. Activation of Ras in dendritic spines [99, 100] is linked, presumably via classical Ras effectors such as extracellular signal-regulated kinase (ERK) mitogen-activated protein (MAP) kinases [101] and phosphatidylinositol (PI) 3-kinases [102], to synaptic remodeling [103]. This remodeling is multifactorial: local modulation of the actin cytoskeleton, which drives morphological changes [104]; altered protein trafficking, which leads to potentiation of AMPA-type glutamate receptors [105]; and changes in transcription [106] and translation [107, 108]. While Ras proteins have the potential to drive all these events, other members of the Ras superfamily may also play important roles. For example Rap, which is linked to control of AMPA receptor trafficking [105, 109], and Rac [110], which is linked to control of the actin cytoskeleton [111], particularly via p21-activated kinase (PAK) [112], are also likely to be important in synaptic remodeling. 

There is substantial evidence that there may be a specific role for the H-Ras isoform in control of synaptic plasticity. Costello syndrome, which has germline expression of activated H-Ras, includes neurological deficits [113]. Further, induced expression of constitutively-active H-Ras.V12 in pyramidal neurons induces neuronal remodeling that is reflected in increases in the number of dendritic spines [114] and synaptic connectivity [115], while the H-Ras knock-out mouse has altered long-term potentiation (LTP) [116]. Activated H-Ras also directs a presynaptic pathway that drives learning and memory [117]. 

H-Ras, as compared to K- and N-Ras, exhibits high expression levels in the neocortex, hippocampus, striatum, thalamus, and cerebellum, in an expression pattern similar to the GEF called Ras-GRF1 [24]. H-Ras is the selective *in situ* substrate for Ras-GRF1 [118, 119]. Ras-GRF1 is highly expressed in certain CNS neurons [120, 121], and is particularly enriched in postsynaptic densities [122], where it can bind directly to the NMDA NR2B receptor and mediate its activation of ERK [123]. Ras-GRF1 can also integrate signals from the modulatory G protein-coupled receptors that also control learning and memory [11, 124–126]. Mice deficient in Ras-GRF1 have learning deficits that include defects in hippocampal-dependent contextual memories [127, 128] and sensory memory formation [129]. Interestingly, the Ras-GRF1 gene is at a susceptibility locus for myopia [130]. Detailed analysis has revealed abnormalities in LTP [131], long-term depression (LTD) [132], neuronal excitability [133], and gene expression [134] in Ras-GRF1-deficient mice. Overall, in light of the increasing concern for the negative effects of traditional cancer chemotherapeutic treatments on cognition [135] and the possibility that targeted therapies will be most effective when combined with traditional modalities [136], the potential for Ras-directed therapy to also have an impact on learning and memory could be significant.

## 7. Targeting of Ras in Type 1 Neurofibromatosis (NF1)

Type 1 neurofibromatosis (NF1) is an autosomal dominant disorder that, with a birth incidence of approximately one in 3000, represents the most commonly inherited predisposition syndrome for both cancer and neurological problems [137, 138]. In addition to a variety of cutaneous, neurological, endocrine, cardiovascular, and orthopedic manifestations, patients typically develop one or more neurofibromas on their peripheral nerves [139]. Benign neurofibromas are tumors that arise in nearly all NF1 patients and have been classified into dermal and plexiform types [140]. Even though dermal neurofibromas do not represent an enhanced risk of progression to malignancy, they can impose a significant burden on the quality of life of NF1 patients. Plexiform neurofibromas are often large tumors that bear the additional risks of potential nerve compression leading to paralysis and progression to malignant peripheral nerve sheath tumors (MPNSTs) that are a main cause of mortality [141]. 

NF1 is caused by loss of expression of the tumor suppressor neurofibromin [142, 143], which is a GAP and potent negative regulator of Ras. Loss of neurofibromin compromises Ras inactivation and leads to constitutively active Ras-dependent signaling [144, 145]. In view of the rationale that NF1 is likely to be driven through inappropriate Ras activation, the FTI tipifarnib was tested in phase II clinical trial for pediatric NF1 patients with progressive plexiform neurofibromas, but positive responses have not been reported. In view of reports that human NF1 tumor cells exhibit constitutive activation of N- and K-Ras, but little or no H-Ras [146], and that the N-Ras activation in NF1 tumor cells drives ERK MAP kinase activation and transcriptional reprogramming [147, 148], it may not be surprising that tipifarnib did not produce significant benefit. Overall, the definition of the pathways that are driven by neurofibromin loss and N-Ras activation in NF1 tumors may reveal effective targets that will improve the treatment options for patients [149].

In addition to the prevalent cases of tumors and risk of cancers in NF1, there is an approximate 50% incidence of pediatric learning disorders [150]. In light of the previous discussion of a role for H-Ras in the neurobiology of learning and memory, it is possible that central dysfunction of H-Ras underlies at least some of the neurological difficulties of NF1 patients, even if H-Ras may not be driving the tumors that arise peripherally in NF1. For example, the learning deficits in a mouse model of NF1 can be ameliorated by treatment with an FTI or genetic reduction of Ras activity [151]. More surprisingly, since levels of statins that will impair the prenylation of proteins are hard to achieve *in vivo* [152, 153], lovastatin also improves cognitive function in a mouse model of NF1 with a mechanism proposed to be through decrease in Ras function [154]. Lovastatin has entered clinical trial for pediatric NF1 patients based upon this rationale [155].

## 8. Other Mechanisms to Target Ras Maturation

After prenylation, the next two postprenylation processing steps of Ras processing are proteolytic cleavage of the carboxyl-terminal “aaX” tripeptide by Rce1 and methylation of the resulting carboxyl terminus by Icmt. In theory, inhibition of these steps might target prenylated variants of all Ras isoforms (both those that are farnesylated through the physiological pathway and those that are iatrogenically geranylgeranylated in the presence of FTIs) and so could provide alternative approaches to inhibit Ras activity. However, much less is known about the mechanism of action of these enzymes as compared to FTase, and the role of the methyl group in protein localization and function is also unclear, and so the effects of inhibition are even less predictable. 

Young and coworkers have shown that targeted inactivation of the *Icmt* gene in mouse fibroblasts causes striking mislocalization of K-Ras [156, 157]. Oncogenic Ras transfected into these cells was not able to support cellular transformation [156, 158]. Furthermore, oncogenic Ras transfected into *Icmt* ^−/−^ embryonic stem cells inhibited cellular transformation, suggesting a crucial role for the methyl group [156, 159]. Additional studies confirm that methylation is required for the proper localization of Ras, but not for localization of the Rho proteins, a class of CaaX proteins that are modified by geranylgeranylation [160]. This differential effect was linked to the fact that Ras is farnesylated and the Rho proteins are geranylgeranylated, which would suggest that Icmt inhibition will actually have a much more profound inhibitory effect on the activity of farnesylated proteins. Thus it may be conjectured that inhibiting Icmt will result in a phenotype similar to that observed when inhibiting FTase alone. Taken together, these data suggest that (i) inhibition of Icmt should lead to the mislocalization of farnesylated Ras but may not have detrimental effects on the function or localization of geranylgeranylated CaaX proteins; (ii) this mislocalization of Ras should interfere with its biological activity; and (iii) Icmt inhibitors are intriguing potential anticancer agents but might suffer from a similar limitation of activity due to the alternative prenylation pathway for K- and N-Ras. In this regard, a selective Icmt inhibitor (cysmethynil) blocks the growth of colon tumor cells in an Icmt-dependent manner [161] and reduces growth of PC3 prostate cancer cell xenografts [162]. 

Some progress has also been made toward selective Rce1 inhibitors [163]. Overall, however, the effects produced by these inhibitors may not be related to effects on Ras, as opposed to other CaaX-containing proteins [12], and it is not clear why they would be any more clinically useful than the FTIs. Similar limitations in our lack of understanding of the detailed enzymology and function, plus the additional uncertainty provided by the reversibility of the modification, would apply to consideration of whether palmitoylation [164] could be blocked to decrease Ras activity.

The final step in Ras maturation is its insertion into the appropriate membrane locale for its functional activity. There is increasing evidence that the spatial organization of Ras proteins is tightly regulated [165]. New information on this topic continues to emerge and clustering of Ras has been proposed to be a target for intervention [166]. The insertion step has been targeted by compounds based on the farnesyl isoprenoid structure, farnesylthiosalicylic acid/salirasib [167], and TLN-4601 [168]. Both of these drugs have entered single agent clinical trials, but the former was inactive against K-Ras mutation positive lung adenocarcinoma [169], and the latter was inactive against progressive glioblastoma multiforme [170]. On the other hand, the results from a combination study of salirasib with gemcitabine for pancreatic adenocarcinoma were interpreted as sufficiently encouraging to warrant further investigation [171]. 

## 9. Indirect Targeting of Ras at the Level of Expression

The difficulty of directly targeting the activity of the Ras proteins themselves has produced a continuing interest in whether their expression level could be reduced as an alternative means to block their function. For example, early efforts pursued antisense approaches to inhibit expression of particular isoforms [172], with notable consequent reversion of malignant phenotype *in vitro* [173]. However, translation of antisense reagents into the clinic has been difficult. In the context of cancer treatment, most progress was apparently made by oblimersen (which targets Bcl-2), but that has apparently stalled prior to confirmation of efficacy and approval [174]. Development of antisense approaches to Ras may have stopped some years ago [175]. 

Regulation of the transcription and translation of Ras isoforms is under endogenous regulation that has also been proposed to provide potential opportunities for intervention. For example, the promoter of K-Ras includes a guanine-rich sequence that can form a G-quadruplex structure that regulates expression [176]. The presence of such structures in human cellular DNA has recently been confirmed [177]. From the perspective of potential therapeutic intervention it is encouraging that a range of already approved drugs [178] and novel agents [179] can bind to and stabilize G-quadruplex DNA structures. These compounds can thus regulate the expression and activity of Ras in *in vitro* systems [180]. Major limitations on the potential translation of this work into the clinic may stem from the fact that the regulatory activity of G-quadruplex structures on transcription can be negative (for H-Ras) or positive (for K-Ras) [180] and from the increasing recognition that sequences compatible with the formation of G-quadruplex structures are widespread in the human genome [181]. It is hard to envisage how modulation of this DNA structural element could lead to a useful and selective therapeutic action in the context of Ras targeting.

Another endogenous mechanism to regulate the expression of Ras is through the noncoding regulatory RNAs called microRNAs or miRs. This property was first discovered in the let-7 suppression of Ras expression in *C. elegans *[182], with demonstration that such regulation is conserved for human N-Ras, K-Ras [182], and H-Ras [183]. Subsequent work has demonstrated that there can be isoform selectivity in miR action, with miR-18a*, miR-96, and miR-622 all having tumor suppressor activity through decrease of K-Ras expression [41–43]. Expression of particular miRs may also affect clinical outcome and response to therapy in K-Ras-driven tumors [184]. The first clinical trial of a miR agent in cancer has recently begun [http://clinicaltrials.gov/ct2/show/NCT01829971]. They are testing a modified version of miR-34, which would be expected to target the p53 pathway [185]. Whether the miR approach will prove clinically useful and adaptable to selective targeting of Ras is, therefore, currently unknown.

## 10. Targeting Pathways Downstream of Activated Ras

The principal focus of recent efforts at inhibition of Ras function has shifted to the development of selective inhibitors of the downstream pathways that are driven by activated Ras [12]. Validation of this downstream approach is provided by the recent FDA approval of the mitogen-activated protein kinase (MAPK) kinase (MEK) inhibitor trametinib [186, 187]. Notably another MEK inhibitor, MEK162, has shown promising results in patients whose melanoma is positive for mutated N-Ras [188]. 

Despite the very encouraging recent results with MEK inhibitors, it is reasonable to speculate whether intervening in the downstream pathways driven by activated Ras may ultimately prove less effective than the putative ability to target directly at the level of Ras itself. Because of the wide divergence of potential signaling mechanisms driven by activated Ras [189–195], it may be difficult to get effective overall inhibition unless the signal is stopped at the source. When the driving oncogenic signal from activated Ras is transmitted through multiple pathways then presumably effective block will require combinatorial inhibition of all the relevant downstream events. It can be challenging to even define the pathway through which the Ras oncogenic signal is being transmitted *in situ* because the dependence on different pathways can vary with experimental conditions [196]. 

In the event that clinical situations can be identified where the Ras-driven signal may be predominantly through a single pathway, such as that through ERK MAPK that can be selectively blocked with MEK inhibitors, there would presumably be high potential for resistance to develop via selection of a subclone that has oncogenic signaling through, for example, the PI 3-kinase/Akt pathway. In melanoma cells that are driven by mutated N-Ras, combined targeting of both MEK and mTOR, which is downstream of PI 3-kinase/Akt, is required for effective inhibition and actually produces a synergistic benefit in culture and xenograft studies [197]. The best available result, even with an effective drug that had excellent pharmacokinetic and pharmacodynamic properties that allowed selective and durable target inhibition with acceptable levels of toxicity, might therefore be a transient beneficial effect that is truncated due to a switch to another pathway that is not inhibited. While extremely encouraging, the results from targeting within the pathways downstream of activated Ras do indicate that responses may be short lived [188].

The example of targeting melanoma that is driven through oncogenic mutant Raf with the inhibitors vemurafenib and dabrafenib also provides several interesting lessons that are likely to be relevant to any Ras-directed therapy that is developed. First, effective targeting of the driving oncogenic event may produce extremely exciting and dramatic responses in at least a subset of patients [198–202]. Second, that resistance will emerge [203]. Third, that investigation of the patterns of inherent and acquired resistance to the targeted inhibitor will reveal significant new information about even signaling pathways that had been considered to be well characterized [204–208]. Encouragingly, pursuit of this new information will help to guide further iterations of inhibitors and their use that may provide further incremental improvements in patient survival [209–211].

## 11. Conclusions

The recognition that driving, oncogenic Ras mutations are prevalent in human cancer, together with the early studies that identified the molecular defect, gave promise that rationally designed, targeted therapeutics for a broad swath of cancers would follow. The great disappointment that has come from the failure of the FTIs in large-scale, phase III clinical trials has led to a necessary reassessment of the field. At a fundamental level the basic difficulty was recognized very early on: although the oncogenic mutations in Ras produce phenotypic gain-of-function, the actual molecular mechanism is one of loss-of-function in that the system is missing the necessary GTPase activity and GAP interaction. In that regard, activated Ras could be viewed as perhaps as difficult a target as the lost tumor suppressor functions, for example, p53, that are also often prevalent in human cancer and that have proved to be extremely challenging to exploit therapeutically. The barriers to direct targeting of activated Ras contributed to the rationale for inhibition of FTase as a druggable route. It is unfortunate that so much of the preclinical work on FTIs focused on systems with mutated H-Ras and somewhat surprising that the momentum behind FTIs remained so strong that the large clinical trials went ahead on lung, colon, and pancreatic carcinoma even though it was already clear at that stage that K- and N-Ras could circumvent effective FTase inhibition by an alternative pathway of geranylgeranylation. Whether the FTIs might actually be useful for patients with disease that is driven by rarer mutations in H-Ras does not seem to have been systematically investigated but perhaps could result from the growing trend of personalized medicine. 

The failure of the FTIs to show efficacy in the Ras-driven cancers tested has left inhibition of the pathways downstream of Ras as the principal approaches currently in the clinic. It is extremely exciting that trametinib has just received FDA approval as the first-in-class with MEK inhibition as its mechanism of action. There are, however, a multitude of signaling pathways that can be driven by activated Ras, and thus it seems likely that inhibition of any one of these pathways may not lead to durable responses. Overall, effective block of activated Ras itself is still a desirable goal that has proven to be difficult to obtain due to several very significant theoretical and technical hurdles. Thus it is extremely encouraging that there has recently been preclinical progress in targeting of the protein:protein interactions that Ras makes with both its upstream effectors and downstream effectors. If the formidable problems of direct targeting of Ras can be overcome, then effective treatments for a broad range of human cancers could eventually result.

## Abbreviations

CaaX: A carboxyl-terminal motif where C means cysteine, a means any residue (typically aliphatic), and X means a variety of amino acids, particularly serine, methionine, glutamine, alanine, or leucine
CaMKII: Calmodulin-dependent protein kinase II
ERK: Extracellular signal-regulated kinase
FPP: Farnesyl pyrophosphate
FTase: Protein farnesyl transferase
FTI: FTase inhibitor
FPP: Farnesyl pyrophosphate
GAP: GTPase-activating proteins
GEF: Guanine nucleotide exchange factors
GGPP: Geranylgeranyl diphosphate
GGTase I: Protein geranylgeranyl transferase I
Icmt: Isoprenylcysteine carboxyl methyltransferase
LTD: Long-term depression
LTP: Long-term potentiation
MAPK: Mitogen-activated protein kinase
MEK: MAPK or ERK kinase
miR: MicroRNA
MPNST: Malignant peripheral nerve sheath tumor
NF1: Type 1 neurofibromatosis
PI: Phosphatidylinositol.
